# Molecular Detection and Antibiotyping of Multidrug-Resistant *Salmonella* Isolated from Houseflies in a Fish Market

**DOI:** 10.3390/pathogens8040191

**Published:** 2019-10-15

**Authors:** Abdus Sobur, Mehedi Hasan, Emdadul Haque, Asmaul Iqbal Mridul, Ayman Noreddin, Mohamed E. El Zowalaty, Tanvir Rahman

**Affiliations:** 1 Department of Microbiology and Hygiene, Faculty of Veterinary Science, Bangladesh Agricultural University, Mymensingh 2202, Bangladesh; soburvetbau@gmail.com (A.S.); mridul0066@gmail.com (A.I.M.); 2 Department of Medicine, Faculty of Veterinary Science, Bangladesh Agricultural University, Mymensingh 2202, Bangladesh; mmhasan.vet@gmail.com; 3 Faculty of Fisheries, Bangladesh Agricultural University, Mymensingh 2202, Bangladesh; emdadulhaque48565@bau.edu.bd; 4 Infectious Diseases and Anti-Infective Therapy Research Group, Sharjah Medical Research Institute and College of Pharmacy, University of Sharjah, Sharjah 27272, UAE; anoreddin@sharjah.ac.ae; 5 Virology and Microbiology Research Group, Department of Pharmacy, City University College of Ajman, Ajman 18484, UAE

**Keywords:** *Salmonella*, foodborne pathogen, salmonellosis, fish market, vectors, housefly, multidrug, resistance, ciprofloxacin, antibiotype, public health, Bangladesh

## Abstract

Houseflies (*Musca domestica*) are well-known mechanical vectors for spreading multidrug-resistant bacteria. Fish sold in open markets are exposed to houseflies. The present study investigated the prevalence and antibiotypes of multidrug-resistant (MDR) *Salmonella* spp. in houseflies captured from a fish market. Direct interviews with fish vendors and consumers were also performed to draw their perceptions about the role of flies in spreading antibiotic-resistant bacteria. A total of 60 houseflies were captured from a local fish market in Bangladesh. The presence of *Salmonella* spp. was confirmed using PCR method. Antibiogram was determined by the disk diffusion method, followed by the detection of *tetA*, *tetB,* and *qnrA* resistance genes by PCR. From the interview, it was found that most of the consumers and vendors were not aware of antibiotic resistance, but reported that flies can carry pathogens. *Salmonella* spp. were identified from the surface of 34 (56.7%) houseflies, of which 31 (91.2%) were found to be MDR. This study revealed 25 antibiotypes among the isolated *Salmonella* spp. All tested isolates were found to be resistant to tetracycline. *tetA* and *tetB* were detected in 100% and 47.1% of the isolates, respectively. Among the 10 isolates phenotypically found resistant to ciprofloxacin, six (60%) were found to be positive for *qnrA* gene. As far as we know, this is the first study from Bangladesh to report and describe the molecular detection of multidrug-resistant *Salmonella* spp. in houseflies in a fish market facility. The occurrence of a high level of MDR *Salmonella* in houseflies in the fish market is of great public health concerns.

## 1. Introduction

Antimicrobial resistance (AMR) is globally recognized as a serious human health threat. The rapid dissemination of AMR genes is remarkable under selective pressure due to widespread and imprudent use of antibiotics [1]. Various insects, particularly housefly (*Musca domestica*), commonly associated with livestock, poultry, and fish are known to be vectors of AMR genes which can be transmitted to humans [2,3,4]. Therefore, fly-mediated transmission of AMR bacteria is also getting an increasing attention.

Enteric and diarrheal diseases are among the important causes of childhood deaths in developing countries [5]. These diseases are ranked as the second cause of childhood deaths under five years old and are responsible for about 750,000 deaths in this age group of children worldwide [6]. Contaminated food consumption can often cause diarrheal diseases. An assessment of global foodborne disease burden approximated 600 million cases and 420,000 deaths due to foodborne illness in 2010 [7].

The incidence of infectious gastrointestinal diseases is aggravated due to the impact of poor food hygiene standards on public health. This can be exacerbated due to the presence of flies, which serve as vectors for a variety of infectious agents. Different studies showed that flies can carry several infectious agents, such as bacteria, viruses, fungi, and parasites [8,9,10]. Flies may transmit pathogens via their proboscis, hair of body and leg, sticky parts of feet, fecal deposition, or vomitus [11,12]. Houseflies are frequently reported to be the cause of contamination with human pathogens [13,14]. Houseflies are also important pests of humans and livestock globally [15]. Previous studies have showed that the housefly is a vector of *Salmonella* spp. [2,3,16]. In addition, *Salmonella* spp. can survive for up to few weeks in various environments [16]. Flies get optimal conditions for their growth and reproduction due to the lack of hygienic disposal of wastes in food markets. Flies also play a salient role in cross-contamination between dirty environments and food sources as they freely move from one place to another [3].

Food products contaminated with *Salmonella* spp. can cause health hazards and economic consequences [17]. *Salmonella*-contaminated foods may cause diseases such as typhoid fever, paratyphoid fever, and severe food poisoning [3]. Humans are frequently exposed to the pathogen by consuming inadequately cooked or contaminated food. Contaminated environments, water, and close contact with infected animals are among the possible sources of *Salmonella* infection [2].

Typically, *Salmonella* infections are experienced with self-limiting mild gastroenteritis that usually recovers without treatment. However, severe systemic *Salmonella* infections can be life-threatening to children, and antibiotic treatments are required in elderly and immunocompromised individuals [18]. Infections with multidrug-resistant (MDR) *Salmonella* spp. that are transmitted to humans through food are difficult-to-treat diseases [19]. Currently, fluoroquinolones are one of the best drugs of choice for *Salmonella* infections. In addition, tetracyclines have been commonly used in clinical settings in Bangladesh [20,21]. However, drug-resistant *Salmonella* spp. to these two classes of antibiotics are increasing all over the world [22,23,24].

In Bangladesh, fish markets are generally located in open areas where other food shops are also present. Houseflies are very common in fish markets where fishes are cut and the inedible parts of the fish are leftover in the same place. Thus, flies can easily pick up the fish’s gut and surface bacteria that may be MDR, since antibiotics are blindly used in aquaculture in Bangladesh [25,26]. These flies can transmit MDR bacteria to other food shops, particularly ready-to-eat foods, creating serious health hazards to humans. The presence of *Salmonella* spp. in raw Rui fish (*Labeorohita*) and unfrozen Pabda fish (*Ompokpabda*) was previously reported in Bangladesh [27,28]. Moreover, antibiotic-resistant *Salmonella* spp. were also detected in some indigenous fishes in Bangladesh [29]. Therefore, this study was conducted to investigate whether flies persistent in the fish market area carry antibiotic-resistant *Salmonella* spp. that may be of severe threats to public health.

## 2. Materials and Methods

### 2.1. Site Selection and Interviews

Kamal-Ronjit (KR) Market, the central food market of Bangladesh Agricultural University (BAU), Mymensingh (24.727040° S, 90.436593° E), was the place of choice for the present study. The market is popular for supplying a variety of fresh, dry, and processed foods for all the university personnel, including students, teachers, other staff, and to their family members. The study was conducted from March to July 2017. Semi-structured questionnaire-based interviews were conducted with the fish vendors in the market and consumers who bought fish during sampling from the shops. It comprised vendor’s and consumer’s KAP (knowledge, attitude, and practice) about the uncontrolled presence of houseflies in the fish stall, their pathogenic capability, control measures, and intervention methods against flies. Six fish vendors and 29 consumers participated in the interviews.

### 2.2. Sample Size Determination

The present study determined the sample size considering the prevalence of *Salmonella* in houseflies in Bangladesh as 11.11% [30]. The sample size was calculated according to the following formula, as previously reported [31]: *n* = Z^2^pq/d^2^, where, *n* = desired sample size, Z = the standard normal deviation, usually set at 1.96 at 95% confidence level, p = prevalence (11.11% or 0.111), q = 1 − p = (1 − 0.111) = 0.889, d = precision (10%, so d = 0.10). So, *n* = (1.96)^2^ × 0.111 × 0.889/(0.10)^2^ = 37.90. To adjust nonresponse, 20% more samples were taken and the sample size was = (37.90 + 20% of 37.90) flies = (37.90 + 7.58) flies = 45.48 flies. However, we captured 60 flies from the fish market.

### 2.3. Sample Collection and Processing

Fresh fishes were selected in this study because of their high risk to cause foodborne illnesses [32]. Moreover, fish is an important source of protein consumed by humans in Bangladesh [33]. A total of 60 houseflies were captured form KR market over a six-week period, with a collection of 10 flies on each sampling session in a week. A glass container was used to hold a sterile zip-locked plastic bag placed inside the container. The container was placed in the fish shop, keeping the zip of bag unlocked to allow the fly to enter into the bag. Once a fly entered the bag, the bag was locked. The locked bag was removed from the glass beaker and another sterile zip-locked bag was used for the next fly. The same procedure was followed during sample collection. After collection, samples were immediately transferred to the laboratory in the Department of Microbiology and Hygiene, BAU. The characteristics and morphology of the flies were examined using stereomicroscope to confirm the flies were of the *M. domestica* species by investigating various body parts, including antenna, vein, arista hair, and forehead furrows [34]. Then, the flies were kept inside the glass containers at −20 °C for a couple of hours to anesthetize [35]. Each fly was placed in a separate Eppendorf tube containing nutrient broth (HiMedia, India) and was gently vortexed. Then, the fly was removed and the broth culture was incubated aerobically at 37 °C for 6 h to allow the growth of bacteria associated with the body surface of the fly.

### 2.4. Isolation and Identification of Salmonella *spp.*

A sterile loopful from the cultured broth was used to inoculate an XLD (Xylose-lysine deoxycholate) (HiMedia, India) agar plate and the plates were incubated at 37 °C aerobically for 24 h. Black colonies on the XLD agar were presumptively considered as *Salmonella* spp., and colonies were further identified using Gram’s stain and conventional biochemical properties, i.e., urease, sugar fermentation, methyl-red, Voges–Proskauer, and indole tests, as previously described [36]. DNA extraction was performed using the boiling method as previously described [37]. *Salmonella* isolates were confirmed by PCR method using specific *invA* gene primers [38].

### 2.5. Antibiotic Susceptibility Test

Ten commonly prescribed antibiotics (HiMedia, India) in Bangladesh, i.e., ampicillin (AMP, 2 µg), azithromycin (AZM, 15 µg), chloramphenicol (C, 10 µg), ciprofloxacin (CIP, 5 µg), gentamicin (GEN, 10 µg), oxytetracycline (O, 10 µg), tetracycline (TE, 30 µg), streptomycin (S, 10 µg), imipenem (IPM, 10 µg), and meropenem (MEM, 10 µg), were selected for antimicrobial susceptibility testing. Antibiogram phenotyping of *Salmonella* isolates was performed by disk diffusion method using Mueller Hinton (HiMedia, India) agar media. McFarland 0.5 standards were maintained for each culture suspension of the bacterial isolates [39]. The results of the test were recorded as sensitive, intermediately sensitive, or resistant according to the recommendations of CLSI [40]. *Salmonella* spp. that were found resistant to multiple antibiotics (at least three classes of antibiotics) were considered as MDR [41].

### 2.6. Molecular Detection of the Resistance Genes

*Salmonella* spp. phenotypically resistant to tetracycline and fluoroquinolone (ciprofloxacin) were further screened for antibiotic resistance associated genes *tetA, tetB*, and *qnrA* by PCR using specific primers as shown in Table 1 [42,43]. Agarose gel (1.5%) was used to analyze the amplified PCR products by electrophoresis. Electrophoresis was done at 100 volts for 25 min in TAE buffer using Mupid-One electrophoresis apparatus (Advance, Japan). Ethidium bromide was used for staining the product, and ultraviolet trans-illuminator (Biometra, Germany) for visualization. DNA ladder of 100 bp (Promega, USA) was used as a molecular weight marker.

### 2.7. Statistical Analysis

All generated data were entered into the Microsoft Excel (Microsoft Corp., VA, USA) spreadsheet and were analyzed using the IBM SPSS Version 22.0 (IBM Corp. Released 2013. IBM SPSS Statistics for Windows, Version 22.0. Armonk, NY: IBM Corp.). Descriptive analysis was performed to compute the frequencies of *Salmonella* spp. and their resistance and data were expressed as number (n) and percentage (%).

## 3. Results

### 3.1. Semi-Structured Interviews

**Figure 1:**
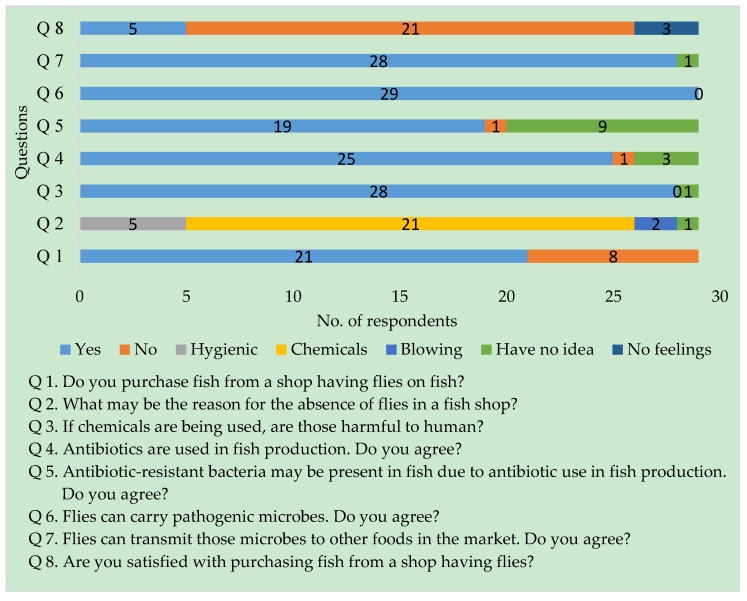
Consumers’ perceptions about flies in the fish market.

**Figure 2:**
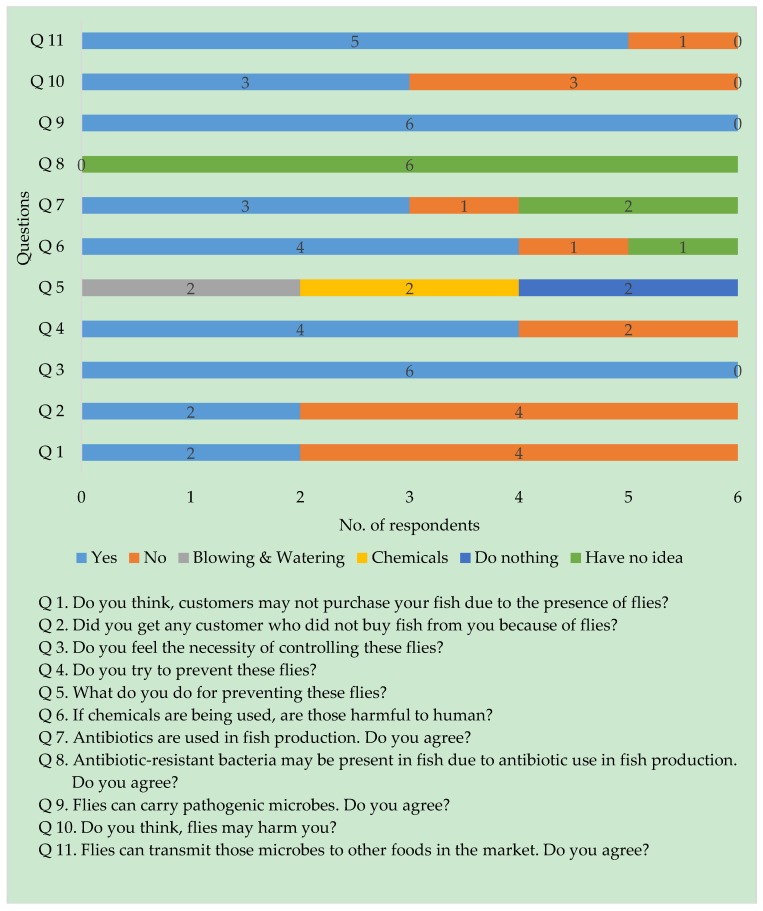
Vendors’ perceptions about flies in the fish market.

Several perceptions were observed throughout the interview (Figure 1 and Figure 2). Among the consumers (*n* = 29), eight (27.6%) reported that they did not buy fish from a shop having flies, and two fish vendors found very few customers who did the same. The rest of the consumers (*n* = 21, 72.4%) bought fishes even if infested with flies, mostly due to lack of shops free from flies, but 66.7% of the 21 consumers were not satisfied due to the presence of flies in the shop. In response to an argument addressed to the vendors whether they realize the necessity of preventing flies in their fish shops, the participated vendors (*n* = 6) agreed that the flies should be controlled in fish markets. Two of the vendors thought to control flies to prevent transmission of diseases, whereas four vendors thought to control flies to create a hygienic and pleasant appearance of their shops.

Among the consumers, 96.6% reported that the use of chemicals to prevent flies in a fish stall may be harmful to humans. On the other side, 17.2% of consumers were satisfied to buy fish from a shop having flies as a sign of no chemical use. Interestingly, 10.34% of the customers had no feelings about satisfaction when considering that both flies and chemicals can cause harm to them.

All consumers and vendors who participated in this study thought that flies can carry and may transmit pathogens to other ready-to-eat foods in the market, except one customer and a vendor, who had no knowledge about the role of flies in the spread of pathogens. Fifty percent of vendors thought that the flies present in the fish market does not cause any harm to them. Among the consumers, 65.6%, who had an educational background at the graduate level, thought that antibiotic-resistant bacteria may present in those flies infesting fish, due to antibiotic use in fish production. On the other hand, all vendors and 31% of the consumers had no knowledge about antibiotic resistance, and only one customer disagreed that antibiotic-resistant bacteria may not be evolved due to antibiotic use. In these circumstances, consumers suggested to prevent flies using a human-friendly method and to control antibiotic use in fish production. In addition, three consumers suggested the application of intervention methods by maintaining proper hygiene and covering the fish with net or glass to prevent disease transmission through flies, and one consumer thought not to use open market and preferred to buy processed fish from supermarkets.

### 3.2. Prevalence of Salmonella *spp.*

**Figure 3:**
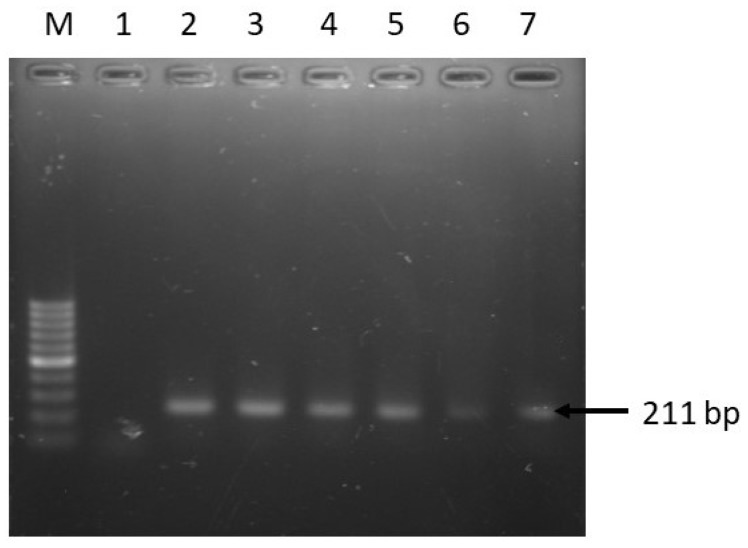
PCR amplification of *invA* gene of *Salmonella* spp. Lane M: 100 bp DNA Marker, 1: Negative control, 2: Positive control, and 3–7: Representative *Salmonella* spp. isolated in this study.

A total of 60 houseflies were captured from the fish market and 78.3% of the cultured samples (*n* = 47) were identified as *Salmonella* based on conventional biochemical tests. However, 34 out of 60 isolates (56.7%) were confirmed as *Salmonella* spp. by PCR method targeting *Salmonella* virulence gene *invA* (Figure 3).

### 3.3. Antibiogram of Salmonella *spp.*

**Figure 4:**
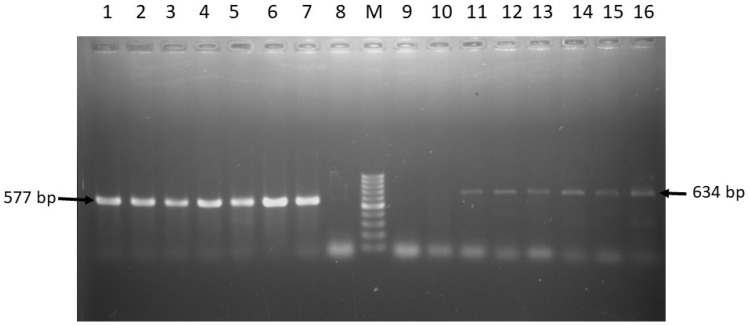
PCR amplification of *tetA* and *tetB* genes of *Salmonella* spp. Lane M: 100 bp DNA Marker, 1–6: Representative *Salmonella* spp. isolated in this study harboring *tetA* gene, 7: Positive control for *tetA* gene, 8: Negative control for *tetA* gene; 9: Negative control for *tetB* gene, 10–15: Representative *Salmonella* spp. isolated in his study harboring *tetB* gene, 16: Positive control for *tetB* gene.

**Figure 5:**
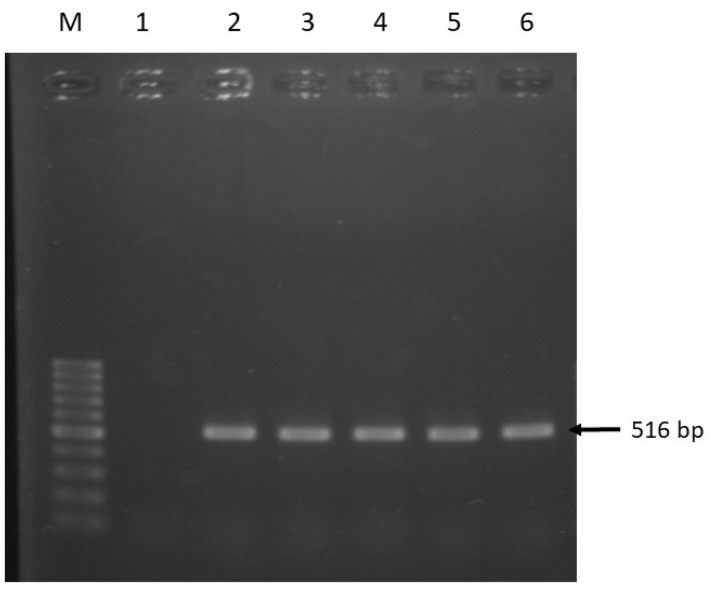
PCR amplification of *qnrA* gene of *Salmonella* spp. Lane M: 100 bp DNA Marker, 1: Negative control, 2: positive control, and 3–6: Representative *Salmonella* spp. isolated in this study.

Antibiogram phenotype test confirmed the isolated *Salmonella* spp. as resistant to multiple antibiotics. The highest observed resistance rate was found against tetracycline (34/34; 100%), followed by ampicillin (28/34; 80%) (Table 2). *Salmonella* spp. isolates that were resistant to ciprofloxacin and tetracycline were further screened using PCR methods to detect the associated resistance genes. As shown in Table 3, all tested isolates that were phenotypically resistant to tetracycline harbored the *tetA* gene, while *tetB* was found in 47.1% (16/34) of the isolates (Figure 4). In addition, about 60% (6/10) *Salmonella* spp. were found positive for *qnrA* gene among those that were phenotypically resistant to ciprofloxacin (Figure 5).

### 3.4. Antibiotyping

Antibiotyping results revealed 25 patterns among the isolated *Salmonella* spp (*n* = 34). (Table 3). There were 23 antibiotypes incorporating 31 (91.2%) isolates that were classified as MDR. Of the antibiotypes, pattern No. 17 (AMP, AZM, MEM, O, S, TE), 20 (AMP, AZM, IPM, MEM, O, S, TE), and 22 (AMP, AZM, CIP, MEM, O, S, TE) were the most prevalent, as each of them had the highest number of three resistant isolates.

## 4. Discussion

Antimicrobial resistance is a major increasing regional and global threat particularly in low and middle-income countries such as Bangladesh where antibiotics are used indiscriminately [44,45]. AMR has the potential to affect almost every sustainable development goal, particularly those targeting hunger, health, poverty, and economic growth [46]. Therefore, AMR is a concern challenging any government. The National Action Plan for AMR of Bangladesh requires baseline data on the occurrence of resistant bacteria and their genes from various sources to develop effective strategies to minimize AMR-related hazards.

In the present findings, the isolation rate of *Salmonella* spp. in houseflies was found to be 56.7%. *Salmonella* spp. detection in flies infesting fish is of great concern due to its potential to cause enteric diseases, which are globally recognized as foodborne zoonoses [7]. It is also important to note that detection of *invA* gene in the isolated *Salmonella* indicates the pathogenic nature of these isolates. *invA* is a virulence gene that encodes an inner membrane protein which is necessary for invasion of epithelial cells [38]. However, it was not unexpected to detect *Salmonella* spp. in flies from the fish market. *Salmonella* spp. are found in the gut microflora and the fish parts that are left over after the fish is processed or cut, and these parts are easily accessible and exposed to the freely moving files. Previously, the detection of *Salmonella* spp. in houseflies infesting fish in food markets was reported in Zambia [3], where a much lower occurrence (7%) of *Salmonella* spp. in houseflies was reported than in the current study. This observed variation might be explained by the differences in the hygienic and sanitary practices in fish markets in Zambia and Bangladesh. To the authors’ knowledge, no report is available from Bangladesh showing that flies in fish markets may carry *Salmonella* spp.

In the current study, houseflies were collected from a fish market, where live birds, goats, and cattle are also slaughtered, processed and the meat products are sold together with fishes—even ready-to-eat foods are also served to humans and disposed of in an open area in the marketplace. Flies carrying *Salmonella* spp. therefore cross-contaminate the surrounding environment during slaughter, cutting, and further processing of fish, meat, or other food products [16,47]. In addition, several studies showed that flies from the environment harboring human pathogens contaminate themselves spontaneously [13,48], thus making the flies more likely to transmit pathogens and cause foodborne illnesses.

Antibiotic administration is important in the treatment of salmonellosis. It is therefore, necessary to determine the resistance patterns of the isolates to prescribe the most effective and appropriate drug. Antibiotic susceptibility pattern results showed that the highest resistance was observed against tetracycline (100%), followed by ampicillin (80%). The observed resistance may be due to the use of tetracycline-like antibiotics in aquaculture [25,26]. Tetracycline-, amoxicillin-, and azithromycin-resistant *Salmonella* spp. were previously detected in fish in Bangladesh [29]. No published data are yet available on AMR-associated houseflies from fish markets in Bangladesh. In a study from Zambia, it was previously reported that all the isolated *Salmonella* spp. were found to be sensitive to the tested antibiotics while a higher resistance rate was observed in the isolated *Escherichia coli* against the tested antibiotics [3]. In the present study, similar to the phenotype, genotypically *tetA* gene was also detected in all *Salmonella* isolates, while *tetB* gene was detected at a relatively lower prevalence (47.1%). These observations are consistent with previous studies which reported that *tetA* and *tetB* genes as the most common genetic determinants of tetracycline-like antibiotics resistance in *Salmonella* spp. [22,49,50,51]. Tetracycline resistance genes can be easily transmitted among various bacterial species, as they are located on mobile genetic elements such as plasmids, transposons, and integrons. Large conjugative resistance plasmids are capable of transferring several resistance genes. These plasmids have been detected in *Salmonella* spp. isolates in several countries and caused cross-resistance to tetracycline [49,50,51].

In the present study, the majority of *Salmonella* isolates (*n* = 31) were classified as MDR, and ampicillin resistance was co-existed simultaneously with tetracycline resistance in 80% of *Salmonella* spp. Meanwhile, among the ciprofloxacin-resistant *Salmonella*, 60% of isolates were *qnrA* positive. The *qnr* genes are plasmid-mediated and have commonly been detected within large conjugative plasmids harboring *tet* genes as well [52]. All of the *Salmonella* spp. that were found positive for *qnrA* in the present study were also found to be positive for *tetA* gene [53]. No report so far is available on the occurrence of fluoroquinolone-resistant *Salmonella* spp. from fish market-associated houseflies from Bangladesh. Fluoroquinolone-resistant *Salmonella* spp. are listed as one of the high priority pathogens by the World Health Organization [54,55]. The occurrence of fluoroquinolone resistance in *Salmonella* isolated from houseflies is therefore of great public health concerns. Moreover, there is a probability of cross-contamination of other food products by flies such as fresh salad, vegetables, fruits, and ready-to-eat food products where the fish is in close proximity, thus increasing the consumers’ health risks.

The semi-structured interviewed respondents’ concerns were justified with our findings over the poor sanitary conditions and the lack of hygienic waste disposal facilities which would facilitate the presence of pathogens as their breeding grounds in the marketplaces. Flies are known to have the potential to spread zoonotic bacterial pathogens because of their free movement from decaying matter, garbage, and feces to human-populated areas [10]. A quick overview of stakeholder’s perceptions about flies in the fish market was observed with the semi-structured interviews. This study found similar observations for flies as a nuisance for the consumers and the fish retailers [3]. However, most of the participants of the present study reported that covering fish with nets or glass for intervention will help in reducing the fly populations in the marketplace. Note should be taken, however, that key decision-makers should be involved in such processes to notify them about the impact of antibiotic resistance on human and animal health so that they can improve sanitary conditions in marketplaces.

## 5. Conclusions

The findings of the current study are of public health importance, as it represents the first report on the occurrence and antibiotyping of multiple antibiotic-resistant *Salmonella* spp. in flies infesting fresh fishes in a marketplace in Bangladesh. The presence of a high level of MDR *Salmonella* spp. detected in the flies captured from the fish market have serious threats to human and animal health. However, it has to be spotlighted that this study was conducted only in a single market; therefore, the results may not completely reflect the overall prevalence of MDR *Salmonella* spp. in the country. Further determination of the serotypes and sequence types, as well as the whole genome characterization of the detected *Salmonella* isolates in this study, will provide important information about the circulating strains and their impact on food safety and human health. Additional hygienic measures, particularly for the appropriate disposal of inedible and unused parts of the fish, should be undertaken to reduce the number of flies in fish selling areas to limit the dissemination of AMR bacteria, thus to ensure improved consumers’ health and safety. In addition, a follow-up pilot study could be carried out to investigate whether the use of glass or nets can reduce the flies from fish stalls, which would limit microbial contamination of food from fish sources.

## Figures and Tables

**Table 1:** Primers used for the detection of *Salmonella* spp. and antibiotic resistance genes.

Target Genes	Primer Sequence (5’-3’)	Amplicon Size (bp)	Annealing Temp. (°C)	Reference
*invA*	F: ATCAGTACCAGTCGTCTTATCTTGAT R:TCTGTTTACCGGGCATACCAT	211	58	[38]
*tetA*	F: GGTTCACTCGAACGACGTCA R: CTGTCCGACAAGTTGCATGA	577	57	[42]
*tetB*	F: CCTCAGCTTCTCAACGCGTG R: GCACCTTGCTGATGACTCTT	634	56	[42]
*qnrA*	F: ATTTCTCACGCCAGGATTTG R: GATCGGCAAAGGTTAGGTCA	516	53	[43]

**Table 2:** Antibiotic resistance of *Salmonella* spp. isolated from houseflies in fish market.

Antibiotics	No. of Resistant Isolates (%)
Tetracycline	34 (100)
Ampicillin	28 (80)
Azithromycin	26 (76.5)
Meropenem	25 (73.5)
Oxytetracycline	25 (73.5)
Streptomycin	23 (67.6)
Imipenem	12 (35.5)
Ciprofloxacin	10 (29.4)
Chloramphenicol	7 (20.6)
Gentamicin	2 (5.9)

**Table 3:** Antibiotyping of *Salmonella* spp. isolates (*n* = 34) and their associated resistance genes.

Pattern No.	Antibiotic Resistance Pattern	No. of Antibiotics (Classes)	Isolate No.^c^.
1	S, TE	2 (2)	41, 50
2	AZM, O, TE	3 (2)	15 ^b^
3	AMP, MEM, O, TE	4 (3)	5
4	MEM, O, S, TE	4 (3)	10
5	AMP, AZM, O, TE	4 (3)	22 ^b^, 51 ^b^
6	AMP, AZM, C, TE	4 (4)	8 ^b^
7	AMP, AZM, IPM, TE	4 (4)	58
8	AZM, C, CIP, O, TE	5 (4)	28 ^a^
9	AMP, AZM, MEM, O, TE	5 (4)	30
10	AMP, C, MEM, S, TE	5 (5)	20 ^b^
11	AMP, IPM, MEM, O, S, TE	6 (4)	14
12	AZM, IPM, MEM, O, S, TE	6 (4)	26
13	AMP, AZM, IPM, MEM, O, TE	6 (4)	35
14	AMP, IPM, MEM, O, S, TE	6 (4)	44 ^b^
15	AMP, AZM, CIP, O, S, TE	6 (4)	55
16	AMP, CIP, IPM, MEM, S, TE	6 (5)	1 ^a^
17	AMP, AZM, MEM, O, S, TE	6 (5)	29 ^b^, 43, 45 ^b^
18	AMP, AZM, CIP, MEM, O, TE	6 (5)	38 ^a,b^
19	AMP, AZM, C, MEM, S, TE	6 (6)	37
20	AMP, AZM, IPM, MEM, O, S, TE	7 (5)	9 ^b^, 21, 24 ^b^
21	AMP, AZM, C, CIP, GEN, MEM, TE	7 (6)	12 ^a,b^
22	AMP, AZM, CIP, MEM, O, S, TE	7 (6)	16 ^a^, 36 ^b^, 52
23	AMP, AZM, MEM, C, O, S, TE	7 (6)	40 ^b^
24	AMP, AZM, C, GEN, IPM, MEM, S, TE	8 (6)	31 ^b^
25	AMP, AZM, CIP, IPM, MEM, O, S, TE	8 (6)	53 ^b^, 57 ^a^

IPM, imipenem; MEM, meropenem; AZM, azithromycin; AMP, ampicillin; CIP, ciprofloxacin; O, oxytetracycline; TE, tetracycline; C, chloramphenicol; GEN, gentamicin; S, streptomycin. ^a^ *qnrA*-positive *Salmonella* isolates, ^b^ *tetB*-positive *Salmonella* isolates, ^c^ all the isolates were *tetA*-positive.
